# It is easy and effective to locate adrenal gland during retroperitoneal laparoscopic left adrenalectomy by the landmark of left PFSV

**DOI:** 10.1038/s41598-023-42269-w

**Published:** 2023-09-13

**Authors:** Ning Wu, Nan Zhang, Jianhuai Chen, Tong Zhao, Songzhan Gao, Jiangbo Zhao, Longfei Lv, Min Lu, Jie Yang, Qinggui Zhong

**Affiliations:** 1 Department of Urology, The People’s Hospital of Jiaozuo, Jiaozuo, China; 2 Drum Tower Clinical Medical College, Nanjing University of Chinese Medicine, Nanjing, China; 3 Department of Urology, The Fifth Clinical Medical College of Henan University of Chinese Medicine, Zhengzhou People’s Hospital, Zhengzhou, China; 4 Department of Andrology, Jiangsu Province Hospital of Chinese Medicine, Affiliated Hospital of Nanjing University of Chinese Medicine, Nanjing, China; 5 Department of Andrology, The Third Affiliated Hospital of Zhengzhou University, Zhengzhou, China; 6 Department of Nursing, The People’s Hospital of Jiaozuo, Jiaozuo, China; 7 Department of Urology, Jiangsu Provincial People’s Hospital, First Affiliated Hospital of Nanjing Medical University, Nanjing, China; 8 Department of Urology, People’s Hospital of Xinjiang Kizilsu Kirgiz Autonomous Prefecture, Xinjiang, Uygur Autonomous Region China

**Keywords:** Nephrology, Urology

## Abstract

To evaluate the feasibility and clinical significance of the left perinephric fat sac vein (PFSV) as an anatomical landmark in locating left adrenal gland (LAD) during retroperitoneal laparoscopic left adrenalectomy (RLLA). In this study, a total of 36 patients who underwent RLLA were enrolled from February 2019 and March 2021. By following a vein vessel on the internal surface of perinephric fat sac (PFS), known as PFSV, LAD was searched finally along the upper edge of this vein. The demographic and clinical characteristics of these patients were acquired, including tumor features and perioperative outcomes (operating time, estimated blood loss, complications). The operations were successfully completed in all the 36 patients without conversion to open surgery. In addition, the LAD was successfully found along the upper edge of PFSV in 34 patients. For all operations, the mean operative time was 75 min (range 60–95) and the estimated blood loss was 20 ml (range 10–50). The median oral intake was 20.7 h (range 6–39). The median hospital stay was 6.3 days (range 4–9), and the median follow-up was 12.3 months (range 9–17). Moreover, no intraoperative complications were observed and no residual tumors were detected after 9 to 15 months follow-up. It may be a safe and efficient procedure to use PFSV as a landmark for searching LAD during RLLA, especially for beginners. However, more studies with larger sample size are need to be conducted to further evaluate the outcomes of this method and the significance of PFSV in searching LAD during RLLA.

## Introduction

With the rapid development of technology, it has become a common belief that minimally invasive adrenalectomy is considered as a gold standard treatment for most adrenal lesion^1–3^. Due to the improvements of advanced equipment and surgical methods, laparoscopic retroperitoneal adrenalectomy is now recognized as a particularly useful method for patients with small benign adrenal tumors, which has many advantages including shorter operating times, less blood loss, avoidance of intra-abdominal adhesion, reduced length of hospital, faster recovery^4,5^.

However, laparoscopic adrenalectomy is considered as a challenging procedure due to the complex anatomical topography of the gland itself, which makes the dissection of adrenal gland somewhat inherently difficult^6^. The adrenal gland is located in a narrow and separate adrenal space, which is triangular, margined with prerenal fascia anteriorly, crus of diaphragma medially, and lamella of the renal fascia laterally. Despite the technical simplicity, factors may defy attempts to localize the adrenal gland, such as the limited working space and the paucity of clear anatomic landmarks^7^. It’s worth noting that the left adrenalectomy is traditionally considered more complex and difficult as the left adrenal gland is positioned beyond the upper pole of the kidney anteriorly^8^. As mentioned by Suzuki^9^, how to identify adrenal gland buried in the perinephric fat was indeed difficult for retroperitoneal adrenalectomy, especially for small tumors. Therefore, the operation time is often significantly prolonged by hemorrhage when surgeons, especially young and inexperienced resident physicians intend to identify adrenal gland in the adrenal space.

During performing retroperitoneal laparoscopic left adrenalectomy (RLLA), we found a new vein between the internal surface of the perinephric fat sac (PFS) and the parenchymal surface of the upper left renal pole, and successfully found the left AD along the upper edge of the vein in fat piles. We believe that the application of the left perinephric fat sac vein (PFSV) as a landmark to search for left adrenal gland is of great clinical significance in RLLA, especially for young surgeons.

## Patients and methods

### Patients

From February 2019 to March 2021, 36 patients at Jiaozuo Hospital who underwent RLLA were enrolled. The indications for RLLA were functioning or non-functioning left adrenal tumors (< 3 cm) without invading surrounding structures. The main clinical symptoms for all patients included hypertension and hypokalemia. Computerized tomography was scaned routinely for all patients and magnetic resonance imaging was performed for some patients as necessary. The preoperative endocrine examination and serum electrolyte levels were routinely evaluated in all patients. Written informed consent regarding potential surgical risks was signed by each patient. The study was approved by the Ethics committee of Jiaozuo People’s Hospital and all methods were performed in accordance with the relevant guidelines and regulations.

### Operative procedures

**Figure 1:**
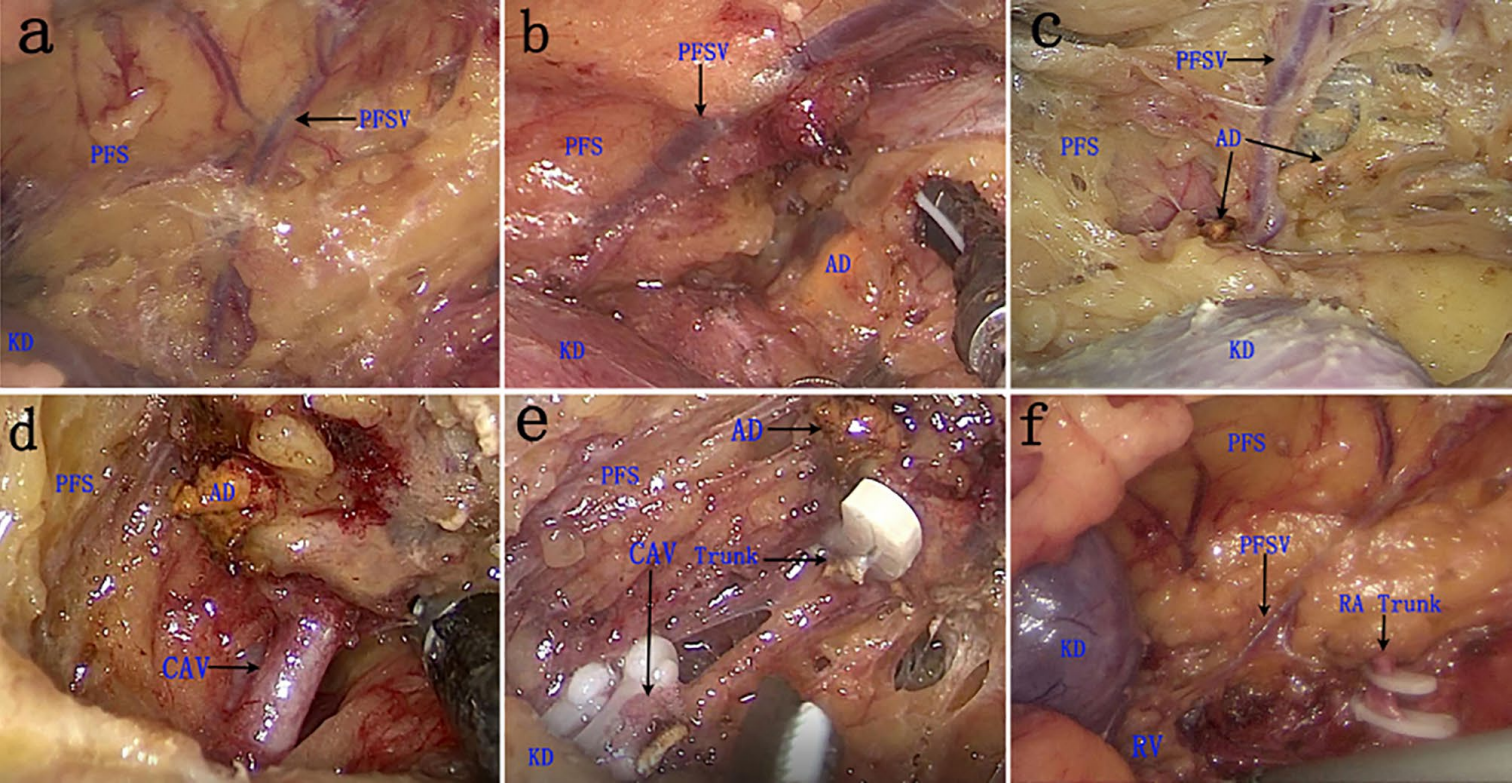
Key procedures of the PFSV and CAV trunk during retroperitoneal laparoscopic left adrenalectomy and the main positional relationship of intraoperative anatomical structures to each other. (**a**) The PFSV trunk, the surface of the upper kidney, and the inner surface of PFS; (**b**) The lateral margin of golden AD is visible when the upper edge of PFS is incised; (**c**) The perirenal fat of AD was completely separated; (**d**) The positional relationship between CAV and the lower pole of AD; (**e**) The CAV was ligated with three Hem-o-lock clips; f The PFSV emanates from the left RV and travels upward into the direction of the diaphragm. *PFSV* perinephric fat sac vein, *PFS* perinephric fat sac, *AD* adrenal gland, *CAV* central adrenal vein, *KD* kidney, *RA* renal artery.

During the RLLA operation, patients were administrated general anesthesia and secured in an overextended full lateral decubitus position with the left side up. The pneumoperitoneum pressure was maintained at 12–15 mmHg. RLLA was performed with the standard 3-port technique as described by Xu et al. in 2007^10^. After retroperitoneal space was established, the retroperitoneal adipose tissue was first removed to the iliac fossa. We identified Gerota’s fascia, cut it vertically from the inferior margin of the diaphragm to the upper margin of the renal, and then the perinephric fat appeared. Afterward, we incised it along the lateral aspect of the PFS to the surface of the renal parenchyma, separated the upper, anterior, medial, and posterior upper fat of the kidney. When the kidney was pressed to the dorsal side, we could see that there was a thick vein on the inner surface of the PFS above the upper pole of the kidney (Fig. 1a). We subsequently dissected the PFS along the upper edge of this vein, and the peripheral contour of the golden adrenal gland emerged (see Fig. 1b,c). Along the lateral border of the adrenal gland, we separated down to the inferior pole of the adrenal gland, and the central adrenal vein (CAV) could be found when the upper part of the inferior adrenal pole was bluntly dissected with an aspirator (see Fig. 1d). We skeletonized the CAV, and ligated it with three Hem-o-lock clips and divided (see Fig. 1e), then completely removed the adrenal gland. The isolated adrenal or tumor was placed in a specimen bag and retrieved. The retroperitoneal space was then reinflated with carbon dioxide gas under a low pressure of 5 to 6 mmHg, and complete hemostasis was confirmed. The adrenal fossa was disposed of the drainage tube. Each trocar was removed under the laparoscopic observation and the incision was sutured.

## Results

The operations were successfully completed in all the 36 patients without conversion to open surgery. No significant complications occurred, including injury to adjacent organs and major vessels. Among all resected tumors, non-functional adrenal adenoma in 5 patients, primary hyperaldosteronism in 5 patients, Cushing’s syndrome in 21 patients and pheochromocytoma in 5 patients (Table 1). The mean size of the resected mass was 2.7 cm (range 1.8–3.0). Median operative time was 75 min (range 60–95). Median intraoperative blood loss was 20 ml (range 10–50). Median oral intake was 20.7 h (range 6–39). Median hospital stay was 6.3 days (range 4–9). Median follow-up was 12.3 months (range 9–17), and no patient received blood transfusion in the postoperative setting. No evidence on imaging of ipsilateral recurrence of adrenal masses was observed (Table 2).

**Table 1:** Demographic and clinical data of patients.

Gender (%)	
Male (%)	19 (52.8%)
Female (%)	17 (47.2%)
Age (years)	48.58 (36–66)
BMI (kg/m^2^)	26.8 (18.7–37.8)
Preoperative diagnosis	
Primary hyperaldosteronism	5
Cushing’s syndrome	21
Nonfunctional tumor	5
Pheochromocytoma	5

**Table 2:** The intraoperative and postoperative outcomes of patients.

Intraoperative outcomes	
Operative time (mins)	75 min (60–95)
Estimated blood loss (ml)	20 ml (10–50)
Resected mass (cm)	2.7 (1.8–3.0)
Peritoneal tear	0
Conversion	0
Postoperative outcomes	
Oral intake (hours)	20.7 (6–39)
Hospital stay (days)	6.3 (4–9)
Followup (months)	12.3 (9–17)

In 34 of the 36 patients, we found PFSV (Table 2), and according to the landmark, we rapidly located the left adrenal gland or tumor. In the remaining two cases, due to the huge volume of the large tumors, the position of the PFSV changed. However, precisely because of the large tumor volume, we could directly see the tumors when the third plane between the perirenal fat and parenchymal surface of the upper renal pole was entered.

## Discussion

In the past two decades, due to the improvements of laparoscopic instruments and surgical techniques, the retroperitoneal adrenalectomy has been favored by increasing surgeons. Since the advent of RLA, surgeons have tried a variety of methods to locate the adrenal gland, and each has its own merits and demerits. Three main ways are advocated for most urologists. The conventional procedure of the adrenal gland often starts with the successive dissection of the renal hilum, renal vein, CAV, and then to the adrenal mobilization, which is called antegrade approach by Walz (Gasman)^11,12^. One of the difficulties of this method is the surgical dissection of renal pedicle, which is resulted from anatomic variations of the adrenal vein in 5% of patients^13^ and, more frequently, of the renal pedicle itself, because an upper pole artery is present in 7% of patients, increasing the risk of vascular injury during dissection^14^. Moreover, the control of adrenal vein is usually difficult when the adrenal gland is not totally dissected. In a series of 500 patients who underwent laparoscopic retroperitoneal adrenalectomy, conversion was necessary in 11 patients (2.2%) due to uncontrollable bleeding^15^.

Another adrenalectomy method, using a retrograde approach according to the technique of Walz et al.^16^, is started by reaching the adrenal gland directly without dissecting the renal pedicle in the first place. The adrenal vein is finally divided. Nevertheless, the adrenal gland needs to be identified by its characteristic color within the adrenal space. Due to the lack of anatomic landmark, it is not easy to identify the adrenal gland in the perinephric fat.

In 2007, anatomical three-level retroperitoneal laparoscopic adrenalectomy was reported by Xu et al.^10^, which has been widely accepted by most urologists due to its safety, efficacy, and treatment outcomes. According to this technique, the adrenal gland can be identified on the outer surface of perirenal fat at the initial stage of the operation when dissecting the first plane between the perirenal fat and anterior renal fascia located at the superomedial side of the kidney. However, the first plane is in anterior side of the kidney and separated by an extremely thin layer of Gerota’s fascia and perotineum, which can lead to peritoneal or even adjacent abdominal organ damage if the operation is slightly improper. In addition, for patients with perinephric adhesion, the completely dissection of the upper pole of the kidney after three-plane dissection can result in nephroptosis in slim patients and time-consuming due to diabetes or a surgical history.

We adopted this surgical method before 2016. To overcome these disadvantages, we modified the anatomic retroperitoneal adrenalectomy and the adrenalectomy was performed through only dissection of the third plane located on the parenchymal surface of the upper renal pole. In our method, we could always see an obvious thick vein on the internal surface of the PFS, and the lateral margin of the golden adrenal gland was visible when we incised PFS along the upper edge of this vein. Since the left CAV was often located in the inner lower part of the left adrenal gland, we could easily find CAV by separating the lower pole of the adrenal gland down along the lateral edge of the adrenal gland.

**Figure 2:**
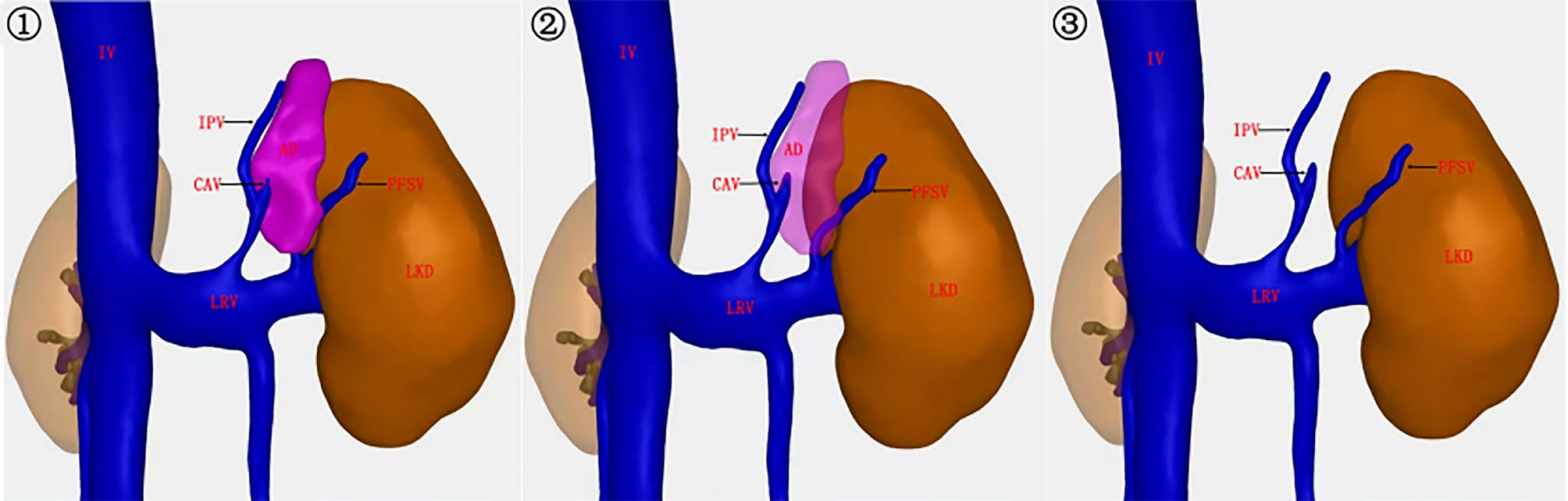
CT three-dimensional (3D) reconstruction stereoscopically showing the location and shape of PFSV. By changing the transparency of the adrenal gland, we could easily identify that the PFSV travels upward along the lateral border of the left AD into the direction of the diaphragm and drains down into the left RV ultimately. *IPV* inferior phrenic vein, *LKD* left kidney, *LRV* left renal vein.

As expected, during a case of laparoscopic nephroureterectomy, we found that this vein converged downward into the left RV and upward deeper into the perinephric fat capsule (Fig. 1f). Meanwhile, we also succeeded in finding this vein through CT three-dimensional (3D) reconstruction (Fig. 2), from which we could easily see that the PFSV emanated from the left renal vein and travels upward along the lateral border of the left adrenal gland and went into the direction of the diaphragm, which demonstrated the routine presence of this vein. We named this vein as “perirenal fat sac vein (PFSV)”.

Some studies reported that the retroperitoneal approach was ideally conducted for masses less than 5 to 7 cm^17,18^. However, Hwang et al.^19^ and Wang et al.^20^ proposed that the retroperitoneal laparoscopic adrenalectomy could be performed in large tumors with a diameter more than 5 cm^5,19^. In our study, the identification of adrenal gland was not difficult when the adrenal tumors were more than 5 cm with routine technique. In this study, all operations were performed smoothly by the same experienced surgeon with our surgical Maneuver and Tactics. These findings give us some inspiration: perhaps one day in the future, the anatomical markers we use will be even smaller blood vessels and tissues that we currently have no understanding of.

IPV sometimes opens into the renal vein. Therefore, any notes regarding the distinction between IPVs that open into the renal vein and PFSVs should be taken seriously. Firstly, our surgical sequence is similar to the retrograde approach. We first found the adrenal gland and then found CAV along the lower edge of the adrenal gland. Therefore, it is not need to determine whether IPVs or PFSVs have both been incorporated into renal V during surgery. Of course, we can also predict the shape of these blood vessels in advance by CT three-dimensional imaging technology before surgery if necessary. Secondly, if both IPVs and PFSVs converge into the renal vein, there are still differences between the two. (1) PFSVs are located on the outer edge of the adrenal gland on the inner surface of the perirenal fat sac and do not significantly penetrate into the perirenal fat sac. On the other hand, IPVs, take shape within the perirenal fat sac and are located at the medial margin of AD. (2) The intersection of PFSVs and renal veins is closer to the kidneys, while IPVs are relatively distant. In our surgical cases, we have not yet found any intersection between the two. Thirdly, in our surgical process, we only rely on PFSVs to quickly locate AD, and then tightly adhere to the lower edge of AD to free CAV, which will appear without the need to continue to free IPVs and PFSVs. This also reflects the advantages of this surgical method in terms of short surgical time and reduced intraoperative free range. Moreover, if both are accidentally removed during surgery, it will not have significant impacts on patients, under the condition that we accurately remove the CAV.

Whether it has the possible utility of PFSV as a landmark in the transperitoneal approach? Due to the fact that retroperitoneal laparoscopic surgery can directly reach the adrenal gland and has the advantages of avoiding stimulation of the abdominal cavity, damage to the intestine, and fast postoperative recovery, our center routinely adopts retroperitoneal adrenalectomy. Generally, laparoscopic adrenalectomy is only performed through the abdominal approach when the patient has a large tumor, or when considering the possibility of malignancy or heavy adhesion around the tumor. Due to the large size of the tumor, it is easy to locate it during surgery without the need for other anatomical markers to assist. In addition, in the intraperitoneal pathway, the renal vein is more easily exposed, and it is relatively easy to find the central adrenal vein along the renal vein, and thus locate the adrenal gland, which is similar to the antigrade approach. That is to say, compared to the retroperitoneal approach, searching for the adrenal gland in laparoscopic adrenalectomy through the abdominal cavity is a relatively mature and easy operation. Of course, the position of PFSV and the adrenal gland within the perirenal adipose sac is not easily affected by other factors such as pneumoperitoneum pressure. PFSV can also be seen theoretically and used to locate the adrenal gland during transabdominal adrenalectomy if we first enter the third layer, namely between the renal pole and the perirenal adipose sac. However, we do not have sufficient experience in this area, and we believe that for surgeons who are accustomed to transabdominal adrenal tumor resection, attempting to use PFSV as an anatomical marker to search for the adrenal gland is also a good choice.

However, there were several limitations in our study. First, it was performed at a single hospital with limited sample size. Although our surgical experience gained from the 36 cases proved our theory, but the limited sample size still made our conclusions not strong enough. Second, since the veins in perirenal adipose capsular had not been studied systematically before, whether the PFSV was variable still needed to be further verified by multi-center, large-sized studies. Third, since we did not detect similar anatomical landmark on the right side, our protocol was just suitable for the left side.

## Conclusion

In conclusion, PFSV may be a useful landmark for finding the left adrenal gland during RLLA, which can shorten operating time and lower conversive rate with very simple operation, and thus is of great clinical significance in RLLA, especially for young surgeons. However, this finding needs to be further verified by multi-center, large-sized studies.

## Data Availability

The datasets used and/or analysed during the current study available from the corresponding author on reasonable request.
